# Coherent X-ray Scattering Reveals Nanoscale Fluctuations in Hydrated Proteins

**DOI:** 10.1021/acs.jpcb.3c02492

**Published:** 2023-05-20

**Authors:** Maddalena Bin, Mario Reiser, Mariia Filianina, Sharon Berkowicz, Sudipta Das, Sonja Timmermann, Wojciech Roseker, Robert Bauer, Jonatan Öström, Aigerim Karina, Katrin Amann-Winkel, Marjorie Ladd-Parada, Fabian Westermeier, Michael Sprung, Johannes Möller, Felix Lehmkühler, Christian Gutt, Fivos Perakis

**Affiliations:** † Department of Physics, AlbaNova University Center, Stockholm University, 106 91 Stockholm, Sweden; ‡ Department Physik, Universität Siegen, Walter-Flex-Strasse 3, 57072 Siegen, Germany; § Deutsches Elektronen-Synchrotron, Notkestrasse 85, 22607 Hamburg, Germany; ∥ Freiberg Water Research Center, Technische Universität Bergakademie Freiberg, 09599 Freiberg, Germany; ⊥ Max-Planck-Institute for Polymer Research, Ackermannweg 10, 55128 Mainz, Germany; # Institute of Physics, Johannes Gutenberg University, 55128 Mainz, Germany; ¶ European X-Ray Free-Electron Laser Facility, Holzkoppel 4, 22869 Schenefeld, Germany; ▽ The Hamburg Centre for Ultrafast Imaging, Luruper Chaussee 149, 22761 Hamburg, Germany

## Abstract

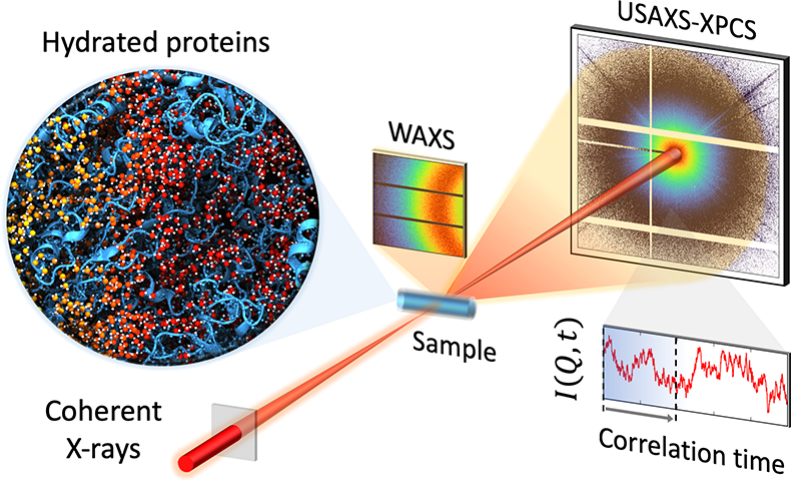

Hydrated proteins undergo a transition in the deeply supercooled regime, which is attributed to rapid changes in hydration water and protein structural dynamics. Here, we investigate the nanoscale stress–relaxation in hydrated lysozyme proteins stimulated and probed by X-ray Photon Correlation Spectroscopy (XPCS). This approach allows us to access the nanoscale dynamics in the deeply supercooled regime (*T* = 180 K), which is typically not accessible through equilibrium methods. The observed stimulated dynamic response is attributed to collective stress–relaxation as the system transitions from a jammed granular state to an elastically driven regime. The relaxation time constants exhibit Arrhenius temperature dependence upon cooling with a minimum in the Kohlrausch–Williams–Watts exponent at *T* = 227 K. The observed minimum is attributed to an increase in dynamical heterogeneity, which coincides with enhanced fluctuations observed in the two-time correlation functions and a maximum in the dynamic susceptibility quantified by the normalized variance χ_*T*_. The amplification of fluctuations is consistent with previous studies of hydrated proteins, which indicate the key role of density and enthalpy fluctuations in hydration water. Our study provides new insights into X-ray stimulated stress–relaxation and the underlying mechanisms behind spatiotemporal fluctuations in biological granular materials.

## 1 Introduction

Proteins undergo a transition upon cooling below *T* ≈ 230 K which impacts their biological function.^1,2^ A signature of a similar transition has also been observed for DNA,^3^ tRNA,^4,5^ and hydrated polymers.^6,7^ Despite the recent progress in the field, the origin of the transition is still a controversial topic and not fully understood. One hypothesis suggests that protein activity is reduced below the transition temperature due to the deactivation of certain degrees of freedom needed for structural transformations essential to protein function.^8−12^ Nuclear magnetic resonance (NMR) studies indicate that motions relevant to protein functionality are activated above 230 K due to the “unfreezing” of hydration water.^13^ This interpretation suggests that hydration water experiences an arrest of collective motion (α-relaxation) upon cooling and that below this temperature, only the local motions are active (β-relaxation).^14^ As a result, this glass-like arrest of hydration water can lead to the deactivation of certain protein degrees of freedom relevant for biological activity. On the other hand, molecular dynamics simulations indicate that cold-denaturation in this temperature range can also lead to impairment of biological function due to protein unfolding.^15−18^ In this case, the low-temperature denaturation occurs due to the disruption of the protein structure, which facilitates the intrusion of water into the protein’s interior and the solvation of buried core hydrophobic residues.

An alternative interpretation proposes that the hydration water is predominantly responsible for the observed low-temperature transition. It is postulated that liquid water exhibits a fragile-to-strong transition at *T* ≈ 230 K as indicated by quasi-elastic neutron scattering (QENS) experiments on hydrated lysozyme powders.^19^ In this scenario, the low-temperature transition is triggered by changes in the hydration water dynamics, which in turn impact the protein activity. This hypothesis is linked to the proposed liquid–liquid transition in liquid water, which suggests a transition from a high-density to a low-density liquid (HDL and LDL).^20,21^ The liquid–liquid transition is hypothesized to take place in the deeply supercooled regime due to the existence of a liquid–liquid critical point.^22^ Simulations and experiments of proteins and other biomolecules in supercooled water indicate that the protein low-temperature transition can be associated with the liquid–liquid transition.^23−25^

Experiments at highly coherent X-ray sources provide a unique opportunity to advance our understanding and gain new experimental insights into collective fluctuations during the low-temperature transition. X-ray photon correlation spectroscopy (XPCS) is a technique that utilizes coherent X-rays and can resolve collective nanoscale dynamics, ranging from microseconds to hours.^26,27^ XPCS has been demonstrated for a broad range of soft condensed matter systems,^28^ including amorphous water where a liquid–liquid transition was observed in the ultraviscous regime.^29^ However, due to experimental difficulties in working with radiation-sensitive samples, XPCS of protein systems became possible only recently with optimized experimental procedures.^30−37^ Previous XPCS studies of lysozyme–water solutions explore the dynamics at room temperature of a pressure-induced liquid–liquid phase separation (LLPS) and viscoelastic coarsening process during gel formation, indicating that simple globular proteins such as lysozyme are capable of forming soft nanostructured protein gels.^37^

Here, we explore the nanoscale dynamics in hydrated lysozyme powders from ambient to cryogenic conditions using XPCS. Hydrated protein powders allow to suppress freezing by confining water in the protein matrix. In this experiment, we combine wide-angle X-ray scattering (WAXS) with XPCS in ultrasmall-angle X-ray scattering geometry (USAXS). This approach provides insights into the previously unexplored low momentum transfer region associated with collective nanoscale fluctuations and stress–relaxation, stimulated by the X-ray beam. The unique advantage of this approach is that it allows to resolve nanoscale dynamics deeply in the supercooled regime (*T* = 180 K), which would normally be frozen in and therefore outside our experimental observation window.

## 2 Methods

### Sample Preparation

The lysozyme protein used is lyophilized powder from chicken egg white purchased from Sigma-Aldrich (L6876). The powder was ground with a mortar to reduce the grain size and used without further purification or drying process. Lysozyme powder was hydrated by exposing it to water vapor in a closed hydration chamber, controlling the humidity and the exposure time to reach the desired hydration value, as characterized previously.^38^ At a hydration level below *h* ≈ 0.3, crystallization is suppressed due to the confinement of water in the protein matrix.^39^ The hydration level for each sample was characterized during the hydration process by measuring the weight before and after hydration. The data shown correspond to an average hydration level of *h* = 0.28 ± 0.05.

### X-ray Experimental Setup

The data were acquired at the Coherence Applications beamline P10 at PETRA III (proposal numbers I-20200072 EC and I-20220280 EC) at the Deutsches Elektronen-Synchrotron (DESY). The measurements were performed simultaneously in ultrasmall-angle X-ray scattering (USAXS) and wide-angle X-ray scattering (WAXS) geometries using a Si(111) monochromator. The experimental results were repeated and reproduced under similar condition; for details on the experimental parameters refer to Table 1. For the two beamtimes, we used different sample environments including a Linkam Stage (model HFSX350) and a liquid-nitrogen coldfinger cryostat in vacuum. For measuring USAXS the Eiger detector was located at a distance of 21.2 m from the sample, while the Pilatus 300k detector was used to capture the WAXS signal. The hydrated protein samples were filled into quartz capillaries of 1.5 mm in diameter and the temperature was controlled in order to have a cooling rate of 5 K/min. By tracking the scattering intensity in WAXS it was possible to monitor freezing of the sample (see Supporting Information). In addition, preliminary XPCS measurements were carried out at beamline ID02 at the European Synchrotron Radiation Facility (ESRF).

**Table 1:** Experimental X-ray Parameters Used for the Two Experiments, Including the Proposal Number, Photon Energy, Beam Size, Flux, Sample Environment, Detector, and Sample–Detector Distance (SDD) for SAXS and WAXS Geometries

experiment	I-20200072 EC	I-20220280 EC
energy (keV)	12.4	9.0
beam size (μm^2^)	30 × 30	30 × 30
flux (×10^9^ ph/s)	4.0	6.0
sample environment	Linkam stage	cryostat
SAXS detector	Eiger 500k	Eiger 4M
SAXS SDD (m)	21.2	21.2
WAXS detector	Pilatus 300k	Pilatus 300k
WAXS SDD (m)	0.21	0.20

## 3 Results and Discussion

### Flux Dependence

**Figure 1:**
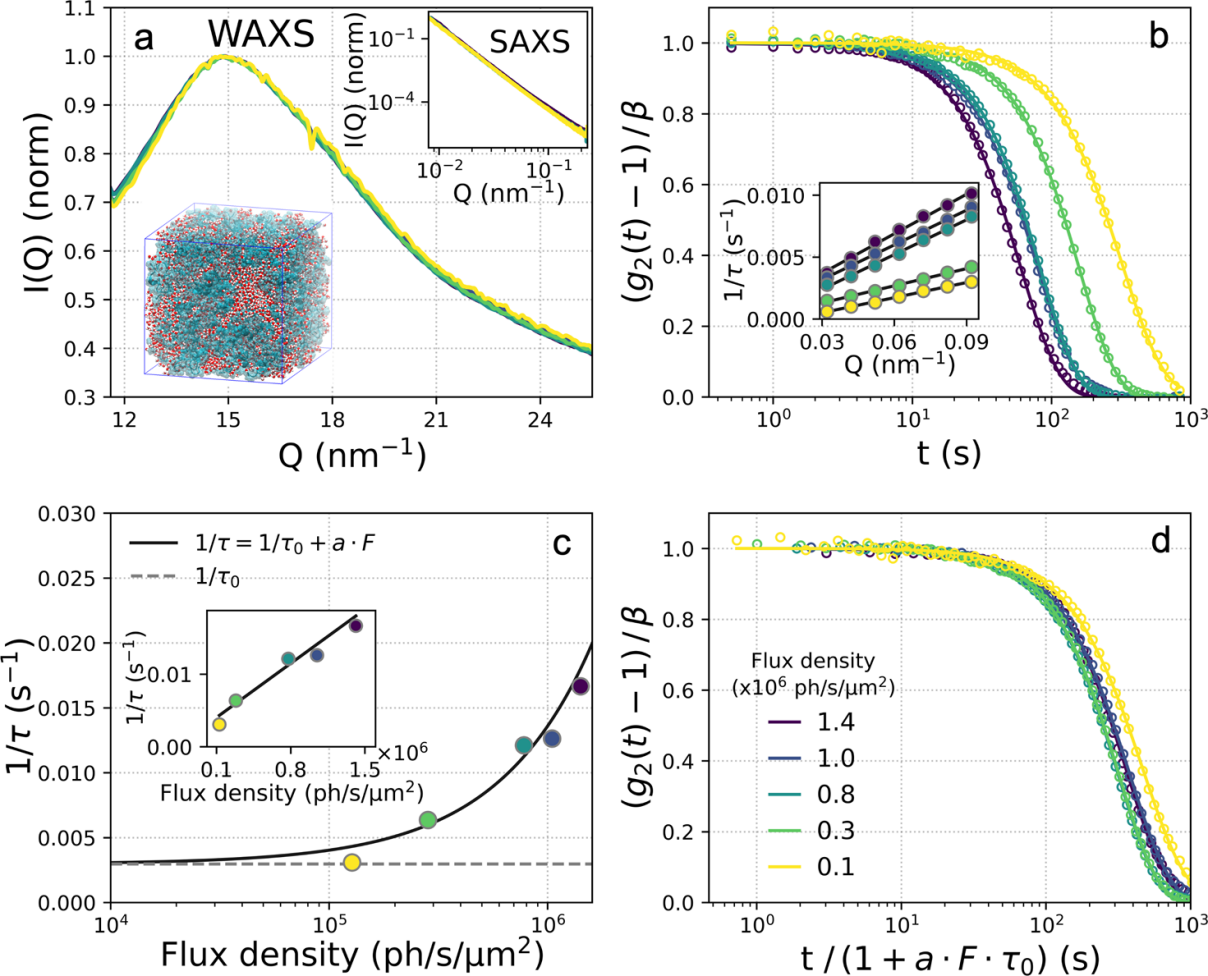
(a) Wide- and small-angle X-ray scattering (WAXS and inset SAXS) intensity as a function of momentum transfer *Q* for different flux densities at room temperature (*T* = 300 K). The schematic depicts the hydrated protein matrix.^38^ (b) Intensity autocorrelation functions *g*_2_ at momentum transfer *Q* = 0.08 nm^–1^ for different flux densities (in units of photons/second/area). The inset shows the linear dependence of the decay rate 1/τ extracted from the fit of the *g*_2_ functions as a function of the momentum transfer Q. (c) The decay rate 1/τ extracted from the fit of the *g*_2_ functions (solid lines) for variable flux density at *Q* = 0.08 nm^–1^. The inset shows the same data on a linear scale. (d) The renormalized intensity autocorrelation functions *g*_2_ at momentum transfer *Q* = 0.08 nm^–1^ for variable flux density, as indicated in the legend. The time axis is normalized to the corresponding flux density *F* by calculating *t*/(1 + *a*·*F*·τ_0_), where τ_0_ is the equilibrium time constant extracted by extrapolation to *F* = 0.

In order to unravel the origin for the observed dynamics we perform flux-dependent measurements at room temperature (*T* = 300 K). Figure 1a shows the WAXS and SAXS intensity as a function of momentum transfer for different flux densities. A minor shift of the momentum transfer *Q* is observed in the WAXS region, whereas no significant changes in the SAXS. In addition, for a given flux density the WAXS signal does not exhibit any significant changes as a function of measurement time (see Supporting Information). Figure 1b shows the temporal intensity autocorrelation functions *g*_2_ at different flux densities indicated in the legend, corresponding from 0% (blue) to 90% attenuation (yellow). The *g*_2_ function is defined as^40^
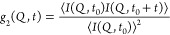
1Here, *I*(*Q*, *t*_0_) and *I*(*Q*, *t*_0_ + *t*) denote the intensity of a pixel at time *t*_0_ and after delay time *t*, respectively. The bracket notation refers to averaging over time *t*_0_ and pixels that belong to a given momentum transfer *Q*-bin, i.e. a thin annulus slice around the beam center corresponding to similar momentum transfers *Q*. The momentum transfer *Q* is defined as *Q* = 4π/λ sin(θ), where λ is the wavelength and 2θ the scattering angle. A stretched exponential function (solid line) is fitted to the resulting correlation functions
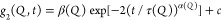
2where β is the speckle contrast, *c* is the baseline, τ is the time constant, and α is the Kohlrausch–Williams–Watts (KWW) exponent.^41^ The obtained *g*_2_ functions exhibit an acceleration of the dynamics for higher flux densities (color-coded in the legend).

Based on the flux density dependence, the observed dynamics are attributed to nanoscale stress–relaxation stimulated by the X-ray beam, which is related to the intrinsic dynamic viscoelastic response of the system to external stimulus. This approach of probing the system response function to external stimuli resembles conceptually the use of cyclic shearing, which can provide information about the dynamic behavior of granular media close to the “jamming transition”,^42^ as well as dielectric spectroscopy which can shed light into the dynamic system response stimulated by external fields near the glass transition.^43^ The time constants extracted from the intensity autocorrelation functions *g*_2_ are inversely proportional to the flux density and resemble ballistic motion, as can be deduced from the observed linear *Q*-dependence of the extracted rate 1/τ (see inset in Figure 1b). Moreover, the time constant τ extracted from the fits as a function of flux density is shown in Figure 1c. The solid line depicts the relation between the extrapolated equilibrium time constant τ_0_ = 336 ± 110 s and the measured relaxation time τ which is modeled by 1/τ = 1/τ_0_ + *a*·*F*. The constant *a* couples the system’s dynamic response to the X-ray beam, which here is estimated from the fit as *a* = (1.1 ± 0.2) × 10^–8^ μm^2^/ph, while *F* is the flux density in (ph/s)/μm^2^. Another similarity to oxide glasses is related to the KWW exponent, which here is α ≈ 1.5 and similar to those obtained for oxide glasses.^44,45^ However, here the probed *Q*-range is not directly sensitive to the local molecular rearrangements but reflects instead the stimulated dynamic response over nanometer length scales. The scattering intensity arises from the density difference in the nanoscale protein grain boundaries and the observed dynamics can be attributed to collective stress–relaxation, as the system converts from a jammed granular state to an elastically driven regime.^46,47^

In hydrated protein-based systems, this kind of dynamic behavior can be potentially influenced by radiation damage attributed to the reaction of proteins with the radicals produced by radiolysis, such as OH radicals.^48^ Such effects however depend on protein concentration as a highly solvent accessible environment is more susceptible to OH radicals^49^ and therefore are more significant in the dilute regime than in hydrated powders. Furthermore, in the present experiment the estimated temperature rise is below 1 K (see Supporting Information) which is consistent with the observed shift in the WAXS^38^ (see Figure 1a), and is insufficient for inducing any changes due to thermal denaturation. The observed dynamics are isotropic (see Supporting Information), except from the presence of streaks due to the grain boundaries that had to be masked as previously.^29^

By normalizing the correlation function *g*_2_ time axis with respect to the flux density shown in Figure 1d, we observe that the curves overlap independent of the flux density used, as seen previously for oxide glasses.^45^ In the current system, however, this extrapolation is limited to room-temperature data; that is because the dynamics in the low momentum transfer range probed here would be too slow and outside the experimental window for the deeply supercooled regime. By stimulating stress–relaxation with the X-rays we are able to obtain information about the nanoscale dynamic response even at cryogenic temperatures.

These results provide new experimental insights into the specifics of the X-ray stimulated dynamics and evolve our understanding of the underlying mechanism. Specifically, the key observations are (a) the time constants extracted from the intensity autocorrelation functions resemble ballistic motion, as deduced from the *Q*-dependence, (b) the KWW exponent measured here is α ≈ 1.5, which is characteristic of driven dynamics, and (c) no major structural changes are observed in the SAXS/WAXS intensity. The observations a and b are consistent with similar features in colloidal gels^50,51^ and polymer melts,^52,53^ as well as in metallic,^54^ oxide,^45^ and network glasses,^44^ providing further evidence of the universality of these concepts. From observation c, we provide indications that beam induced dynamics does not necessarily imply structural changes associated with loss of function, as this phenomenon can occur in elastic systems even below damage thresholds.^51^ This type of dynamical behavior reflects the fact that this class of systems have small elastic moduli, and thus, even moderate forces are sufficient to give rise to strong internal stresses.^55^ Numerical simulations of soft solid systems confirm this picture and indicate that ballistic-type dynamics can occur through intermittent stress releasing events^47^ and local elastic deformations.^56^

### Temperature Dependence

**Figure 2:**
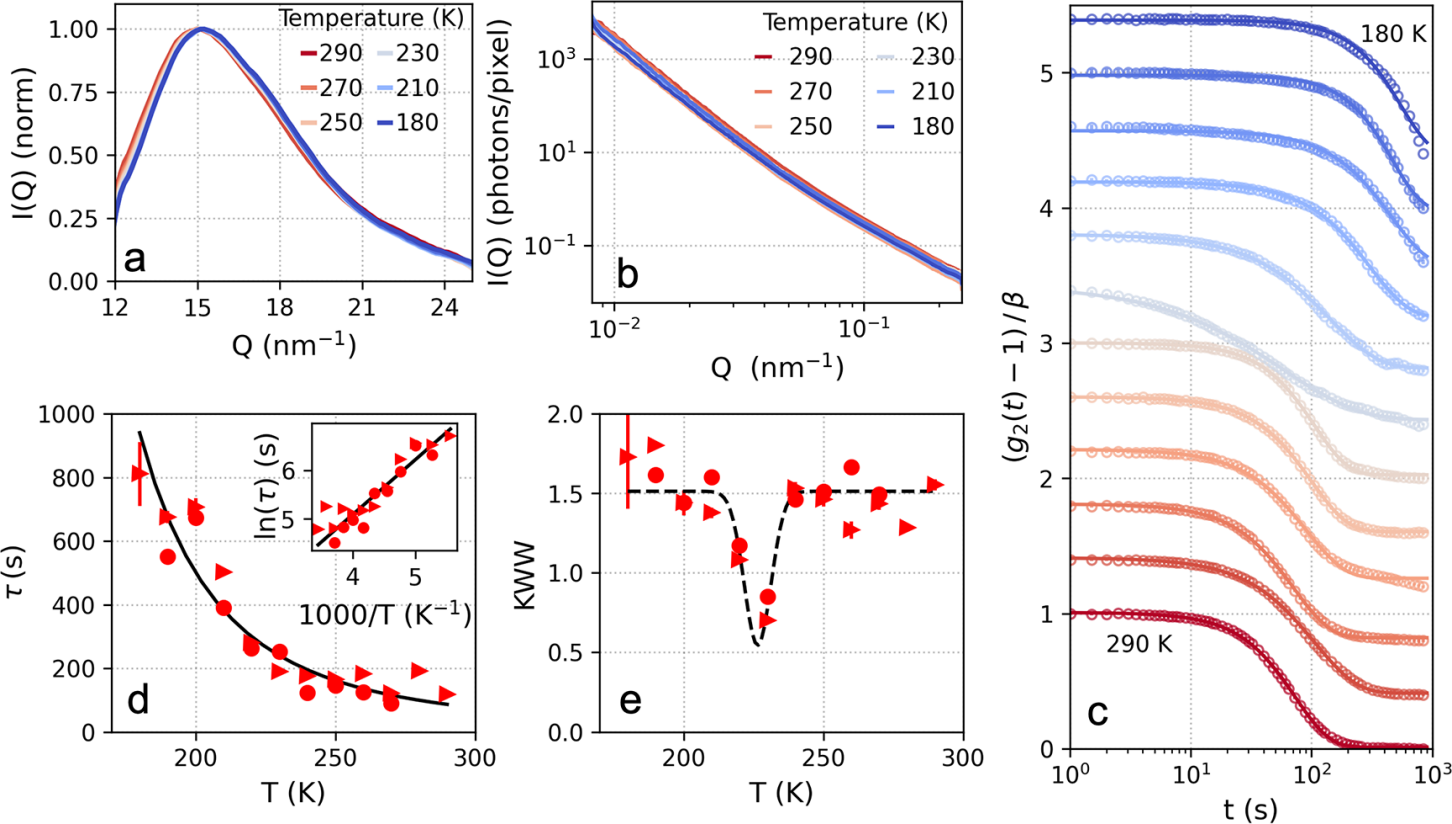
Temperature-dependent measurements. (a) The WAXS and (b) SAXS scattering intensity at different temperatures indicated in the legend. (c) Intensity autocorrelation functions *g*_2_ for different temperatures, upon cooling from *T* = 290 K to *T* = 180 K, as indicated in the plot. The data shown are calculated at momentum transfer *Q* = 0.1 nm^–1^, and the solid lines indicate the fits with a stretched exponential. An offset has been added to facilitate the comparison. (d) The time constants τ extracted from the fits in panel c. The inset shows the logarithm of the time constant τ as a function of the inverse temperature 10^3^/*T* where the solid line indicates the Arrhenius fit. (e) The Kohlrausch–Williams–Watts (KWW) exponent as a function of temperature. The dashed line is a guide to the eye (Gaussian fit) indicating a minimum at *T* = 227 K. The various symbols in panels d and e indicate data acquired during different beamtimes with similar conditions (see Methods).

By recording simultaneously X-ray diffraction data in WAXS geometry along with the XPCS measurements we ensure that the samples have not crystallized (Figure 2a; see also Supporting Information). The observed changes in WAXS intensity as a function of temperature, such as the shift toward larger *Q* upon cooling, agree with previous investigations and indicate temperature-dependent changes on atomic length scales.^38^ We do not observe significant temperature-dependent changes in the SAXS region, as shown in the Figure 2b. In hydrated proteins, the SAXS does not exhibit any peak in the observed *Q*-range as the signal is dominated by the scattering intensity emerging from the grain boundaries and hydrated proteins-air interfaces. This is consistent with previous studies, where it is indicated that for hydrated powders the intensity follows a power law dependence, contrary to solutions that exhibit a peak at *Q* ≈ 1 nm^–1^ due to the protein–protein interactions.^57^ The temperature dependence of the dynamics was measured by using *F* = 1.5 × 10^6^ ph/s/μm^2^ upon cooling from *T* = 290 K down to *T* = 180 K. This experimental condition corresponds to a dose rate of 1.58 kGy/s (see Supporting Information). The intensity autocorrelation *g*_2_ functions are visualized in Figure 2c. The *g*_2_ functions indicate that the system exhibits a slowing down of the dynamics upon cooling.

The extracted time constants τ are shown as a function of temperature in Figure 2d, where the solid line depicts an Arrhenius fit using the relation τ(*T*) = *A*·e^*E*_*a*_/*k*_*B*_*T*^, where *A* = 1.8 ± 1.1 s is the amplitude and *E*_*A*_ = 9.4 ± 1.1 kJ/mol is the activation energy. Additionally, the natural logarithm of the time constants τ are depicted in Figure 2d as a function of the inverse of the temperature (Arrhenius plot, inset). The Arrhenius analysis yields an activation energy which is comparable with that obtained by QENS (13 kJ/mol).^19^ The difference here is that the QENS measurements were performed at a higher momentum transfer region, reflecting local molecular diffusion, whereas the low momentum transfer *Q* probed here reflects nanoscale viscoelastic motion. This difference could explain why here any noticeable crossover in the proximity of the low-temperature transition temperature is not observed, indicated by QENS in hydrated lysozyme powders.^19^ This result is consistent with dielectric spectroscopy measurements, which showed no sign of a transition in the temperature dependence of conductivity^58^ in hydrated lysozyme proteins.

The corresponding KWW exponents as a function of temperature are shown in Figure 2e. We observe that the KWW exponent exhibits a minimum from an average value of α ≈ 1.5 to α < 1 at *T* = 227 K. Similar transitions have been attributed to the emergence of dynamical heterogeneities upon approaching the glass transition temperature,^59−61^ although here we observe that the exponent values return to α ≈ 1.5 below *T* = 210 K. This behavior can also be seen directly from the line shape of the *g*_2_ functions, which appear distinctly more stretched at *T* ≈ 230 K. The observed minimum was reproduced over several beamtimes with similar experimental conditions, indicated by the different symbols in Figure 2, parts d and e (see Methods).

### Two-Time Correlation Analysis

Calculating the two-time correlation (TTC) function^62^ allows us to quantify the dynamical heterogeneity. The TTC is defined as
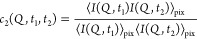
3where *I*(*Q*, *t*_1_) and *I*(*Q*, *t*_2_) denote the intensity of a pixel at distinct times *t*_1_ and *t*_2_. The subscript “pix” implies that, contrary to the *g*_2_ definition, the averaging is, in this case, solely performed over pixels within the same *Q*-bin and not over time.

**Figure 3:**
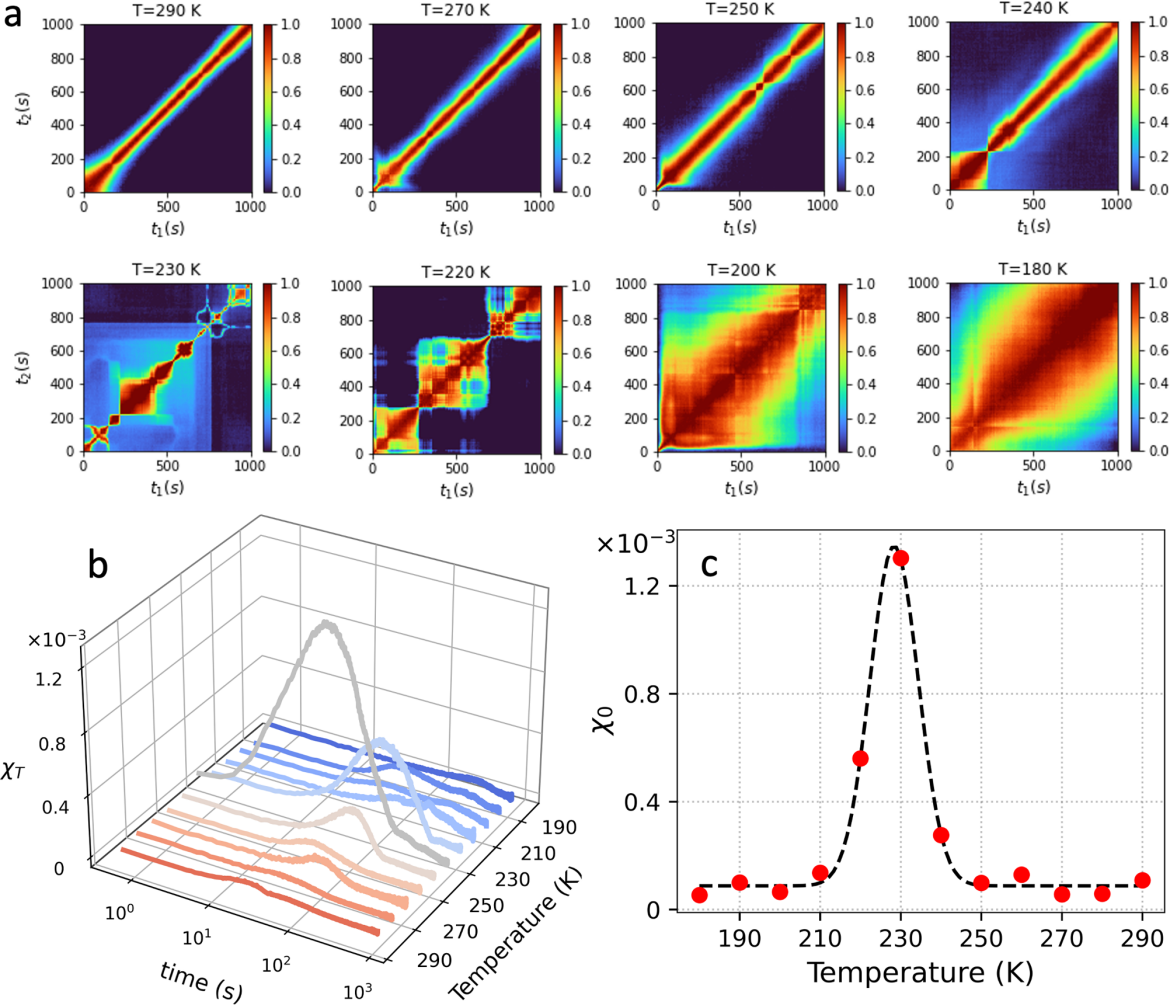
(a) Two-time correlation function (TTC) at different temperatures indicated in the panel titles at momentum transfer *Q* = 0.1 nm^–1^. (b) The normalized variance χ_*T*_ at different temperatures extracted from the TTC. (c) The maximum of the normalized variance χ_0_ obtained at different temperatures indicates a maximum at *T* = 227 K.

In Figure 3a the TTC functions for temperatures ranging from *T* = 290 K to *T* = 180 K are shown. We observe that overall the TTC line shape for higher temperatures looks smooth and continuous. In some instances, an initial acceleration (see, e.g., *T* = 270 K or *T* = 250 K) or deceleration (see, e.g., *T* = 290 K) is observed before the line shape is stabilized, which can be attributed to the initial interaction and stress–relaxation stimulated by the beam. In addition, pronounced fluctuations manifest for *T* = 230 K. These fluctuations are enhanced for the range mainly between *T* = 230–220 K and diminish below *T* = 210 K. It is worth noting that the KWW exponent minimum observed in Figure 2e is a direct consequence of averaging the TTC over multiple correlation times.

The dynamical heterogeneity is quantified by calculating the normalized variance χ_*T*_ of the TTC function, which is an experimentally accessible estimator of the four-point dynamical susceptibility.^63^ The variance is calculated from the TTC, by using the relation
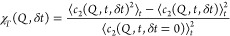
4where *t* and *δt* correspond to the diagonal and antidiagonal axes of the TTC. The subscript *t* implies that the averaging is, in this case, performed over the TTC diagonal axis, as previously.^29^

The χ_*T*_ calculated at different temperatures is shown in Figure 3b. We observe that the amplitude of χ_*T*_ is maximized at *T* = 230 K, consistent with the enhancement of the observed fluctuations in the TTC, which result in distinct peaks in the χ_*T*_ distribution. Interestingly, the mean relaxation time at the χ_*T*_ peak is significantly shorter than the average relaxation time at *T* = 230 K (26 and 220 s respectively), indicating that the observed fluctuations can be attributed to a faster intrinsic dynamic process. The maximum value of the χ_*T*_, denoted as χ_0_ in Figure 3c, exhibits a maximum at *T* = 227 K. This enhancement in χ_*T*_ is an indication of maximization of dynamical heterogeneities in this temperature range which manifests as fluctuations in the TTC function. Such enhancement of the dynamic susceptibility in granular materials has been previously associated with a growing dynamic correlation length due to spatiotemporal fluctuations.^42,64^

The present observations are consistent with previous investigations of the dynamics in hydrated lysozyme, where a maximum in the specific heat capacity was attributed to a sharp change of local order and enhanced hydrogen bond fluctuations.^24,65^ Furthermore, a maximum in the dynamic susceptibility at *T* = 230 K has been associated with growth in size of dynamic heterogeneity in confined water.^66^ The current data are also in-line with the crossing of the Widom line, defined as the locus of points in the *P*–*T* surface, which have a maximum in the correlation length. The specific heat capacity and isothermal compressibility of pure supercooled water exhibit maxima at the Widom line,^20,67^ which are thermodynamic response functions representing enthalpy and density fluctuations. Even though the confinement of hydration water in the protein matrix can influence the local structure^38^ our data here indicate that it is still possible to capture collective fluctuations at *T* = 227 K which correlates with the Widom line temperature at ambient pressure in pure liquid water.^20,67^

## 4 Conclusions

Summarizing, we have studied the stress–relaxation dynamics stimulated by X-rays in hydrated lysozyme powder by using USAXS-XPCS. This approach allows us to obtain information about the nanoscale stimulated dynamic response down to deeply supercooled conditions (*T* = 180 K), which is inaccessible under equilibrium conditions. In hydrated proteins, the scattering intensity arises from density differences in the nanoscale grain boundaries, and thereby, the observed dynamics are attributed to stimulated collective stress–relaxation as the system transitions from a jammed granular state to an elastically driven regime. The observation that the dynamic response clearly fingerprints temperature behavior implies that it couples to the dynamic modes in the system and the stimulated dynamics carry the information about the nanoscale fluctuations. The extracted time constants exhibit Arrhenius temperature dependence accompanied by a sharp minimum in the KWW exponent *T* = 227 K, indicative of dynamic heterogeneity. TTC analysis indicates the presence of pronounced dynamic fluctuations, which are maximized at the same temperature range. The observed amplification of fluctuations at *T* = 227 K is consistent with several studies of hydrated protein powders, which indicate the key role of water.^19,23−25,64,65^ Dielectric spectroscopy experiments in hydrated lysozyme powders, complemented by Monte Carlo simulations, attribute the observed crossover to hydrogen bond fluctuations in the protein hydration water.^24^ Furthermore, NMR spectroscopy studies of hydrated lysozyme powders indicate that the observed maximum coincides with maximum in the heat capacity, which reflects density and enthalpy fluctuations in hydration water.^64^ From a general point of view, our study paves the way for future experiments following nanoscale fluctuations and stress–relaxation in systems where equilibrium dynamics are not accessible with standard methods, such as for granular matter and glassy materials.

## Data Availability

The data that support the findings of this study are openly available in the figshare repository with DOI: 10.17045/sthlmuni.22756400.

## Supporting Information Available

Detection of crystallization and azimuthal dependence of the time constants, as well as on the temperature increase and dose estimations, including additional references (PDF)
