# Supplementary material for: Coherent X-ray Scattering Reveals Nanoscale Fluctuations in Hydrated Proteins
Source: J Phys Chem B. 2023 May 20;127(21):4922–30. doi: 10.1021/acs.jpcb.3c02492 (PMC10240491; doi:10.1021/acs.jpcb.3c02492)
Supplement: Supplementary file 1 — jp3c02492_si_001.pdf [file jp3c02492_si_001.pdf]

# SUPPORTING INFORMATION:
# Coherent X-ray Scattering Reveals Nanoscale Fluctuations in Hydrated Proteins

Maddalena Bin[1], Mario Reiser[1], Mariia Filianina[1], Sharon Berkowicz[1], Sudipta Das[1], Sonja Timmermann[2], Wojciech Roseker[3], Robert Bauer[3,4], Jonatan Öström[1], Aigerim Karina[1], Katrin Amann-Winkel[1,5,6], Marjorie Ladd-Parada[1], Fabian Westermeier[3], Michael Sprung[3], Johannes Möller[7], Felix Lehmkühler[3,8], Christian Gutt[2], and Fivos Perakis*[1]

[1]Department of Physics, AlbaNova University Center, Stockholm University, 106 91 Stockholm, Sweden

[2]Department Physik, Universität Siegen, Walter-Flex-Str. 3, 57072 Siegen, Germany

[3]Deutsches Elektronen-Synchrotron, Notkestr. 85, 22607 Hamburg, Germany

[4]Freiberg Water Research Center, Technische Universität Bergakademie Freiberg, 09599 Freiberg, Germany

[5]Max-Planck-Institute for Polymer Research, Ackermannweg 10, 55128 Mainz, Germany

[6]Institute of Physics, Johannes Gutenberg University, 55128 Mainz, Germany

[7]European X-Ray Free-Electron Laser Facility, Holzkoppel 4, 22869 Schenefeld, Germany

[8]The Hamburg Centre for Ultrafast Imaging, Luruper Chaussee 149, 22761 Hamburg, Germany

*f.perakis@fysik.su.se

# 1 Detection of crystallization

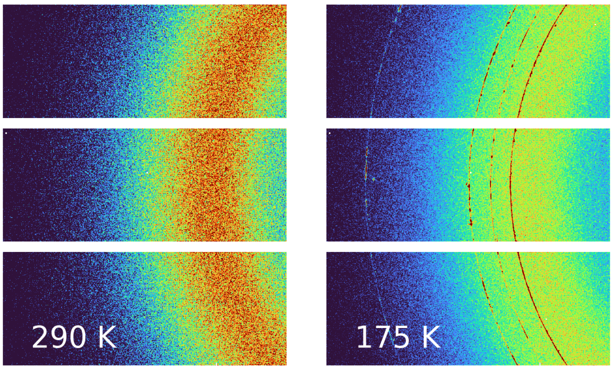


Figure S1: Wide-angle X-ray Scattering (WAXS) 2D intensity for the hydrated proteins at $T = 290$ K (left) and $T = 175$ K (right). The latter shows sharp rings, feature of crystallization.

# 2 Azimuthal dependence analysis

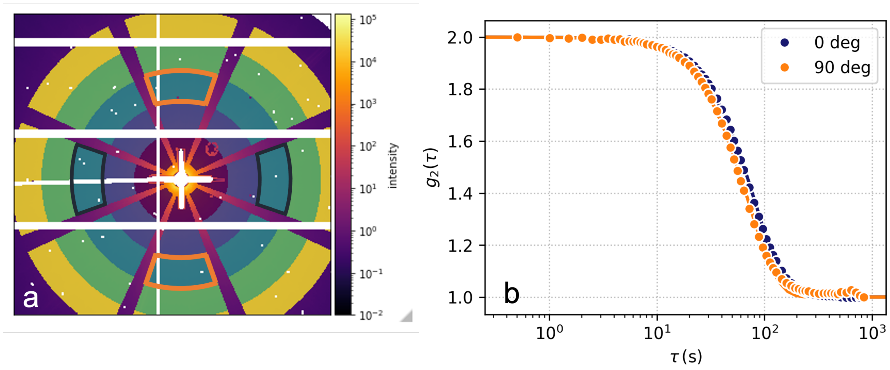


Figure S2: a) Layout of the performed azimuthal dependence analysis. The image depicts the different Q-bins as well as the azimuthal angles. b) The autocorrelation functions $g_2$ calculated for the horizontal component (black) and the vertical component (orange). The corresponding areas in the detector are marked with the same colors in panel a. The analysis shows no major difference between the horizontal and the vertical component.

# 3 WAXS during exposure

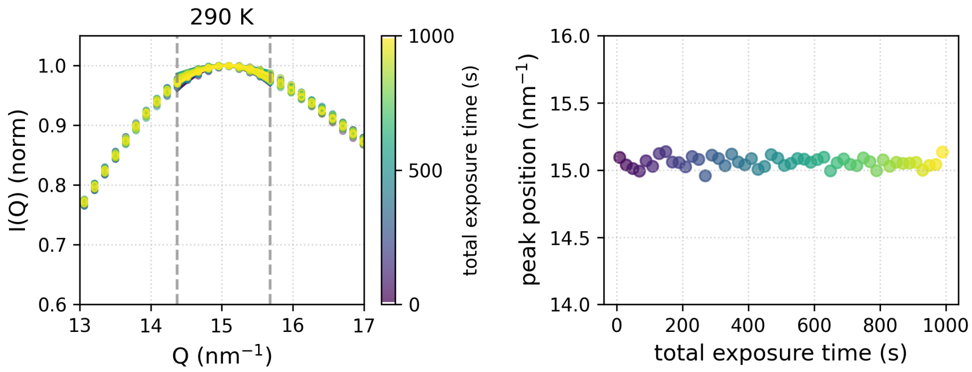


Figure S3: The normalised WAXS intensity I(Q) as a function of momentum transfer Q (left panel) during a measurement with 1000 s exposure time measured with $F = 1.5 \cdot 10^6$ ph/s/$\mu$m$^2$ at temperature T = 290 K. The I(Q) does not show any significant changes and the solid lines depict Gaussian fits used to extract the peak position shown in the right-hand panel.

# 4 Temperature increase and dose estimation

## Temperature increase

The transmission of lysozyme ($T_{\text{lys}}$ = 0.495) for the current experimental conditions (photon energy $E = 12.4$ keV, sample thickness $d_s = 1.5$ mm) is calculated by using the molecular formula $C_{125}H_{196}N_{40}O_{36}S_2$ based on the known atomic data tables [1]. The total sample transmission is estimated by averaging the transmission of lysozyme with that of water ($T_{\text{w}} = 0.673$) weighted by the corresponding mass fraction ($h = 0.3$).

The absorbed energy $dQ$ for a given exposure time $t_e$ is calculated by including the incident beam flux $I$ (in µJ s$^{-1}$) and the sample transmission, which gives $dQ = (1 - T_r) \cdot I \cdot t_e$. This estimation corresponds to a maximum temperature increase of $T_{max}(t) = dQ/m/c_p$ where $m$ is the mass and $c_p$ the heat capacity. Here we used the following values for the lysozyme: isobaric heat capacity $c_p = 1260$ J kg$^{-1}$ K$^{-1}$ [2], density $\rho = 2200$ kg m$^{-1}$ [3] and heat conductivity $k_w = 0.42$ W K$^{-1}$ m$^{-1}$ [4]. The heat dissipation time [5] is calculated by

$$t_0 = c_p \cdot \rho \cdot a^2/(2 \cdot k_w) \approx 3 \text{ ms} \tag{1}$$

where $a$ is the beamsize (30 µm). The corresponding temperature rise [5] is

$$\delta T = \int T_{max}(t) \cdot [1 - \exp(-\frac{1}{[2 \cdot (1 + t/t_0)]})]\mathrm{d}t. \tag{2}$$

This estimation gives values below $\Delta T = 10$ K for the highest flux used here, $I = 4 \cdot 10^9$ ph s$^{-1}$.

## Dose

In order to quantify the amount of energy absorbed by the sample, we calculate the dose $\mathcal{D}$ absorbed by the system by

$$\mathcal{D} = \frac{F \cdot E \cdot A \cdot t_e}{a^2 \cdot d_s \cdot \rho_w}, \tag{3}$$

where $F$ denotes the flux, $E$ the photon energy, $A$ is the absorption, $t_e$ the exposure time, $a$ the beam size, $d_s$ the sample thickness, and $\rho_w$ the weighted averaged density. The values utilized are summarized in Table S1. The calculated dose is $\mathcal{D} = 1.58$ kGy.

The absorption is computed from the transmission as $A = 2 - \log(\%T_{lys})$, with $T_{lys} = 0.495$ (see previous paragraph), which is calculated from atomic data tables and is in agreement with the transmission measurements performed during the experiment. Furthermore, the weighted average density $\rho_w$ is computed based on the mass fraction $h = 0.28$. In particular,

$$\rho_w = \frac{\sum_{i=1}^{n} w_i \rho_i}{\sum_{i=1}^{n} \rho_i} = \frac{w_{lys}\rho_{lys} + w_{wat}\rho_{wat}}{\rho_{lys} + \rho_{wat}} \tag{4}$$

and the weights $w_i$ are calculated from

$$h = \frac{m_{wat}}{m_{lys}} = \frac{\rho_{wat}\, V_{wat}}{\rho_{lys}\, V_{lys}}. \tag{5}$$

By rearranging Equation (5), one obtains that $w_{lys} = 0.73$ and $w_{wat} = 1 - w_{lys}$.

Table S1: Parameters used for the dose estimation

| Quantity | Symbol | Value | Unit |
|---|---|---|---|
| Flux | $F$ | $6 \times 10^9$ | ph/s |
| Photon energy | $E$ | 9 | keV |
| Absorption | $A$ | 0.31 | |
| Exposure time | $t_e$ | 1 | s |
| Beam size | $a$ | $30 \times 10^{-6}$ | m |
| Sample thickness | $d_s$ | $1.5 \times 10^{-3}$ | m |
| Sample density | $\rho_w$ | 1.25 | kg/m$^3$ |

# References


(1) Burton L Henke; Eric M Gullikson; John C Davis X-ray interactions: photoabsorption, scattering, transmission, and reflection at E= 50-30,000 eV, Z= 1-92. *Atomic data and nuclear data tables* **1993**, *54*, 181–342.

(2) Yang, P.-H.; Rupley, J. A. Protein-water interactions. Heat capacity of the lysozyme-water system. *Biochemistry* **1979**, *18*, 2654–2661.

(3) Leung, A. K. W.; Park, M. M. V.; Borhani, D. W. An improved method for protein crystal density measurements. *J. Appl. Crystallogr.* **1999**, *32*, 1006–1009.

(4) Fujiwara, S.; Maki, S.; Maekawa, R.; Tanaka, S.; Hagiwara, M. Measurements of thermal conductivity and thermal diffusivity of hen egg-white lysozyme crystals and its solution using the transient short hot wire method. *Int. J. Thermophys.* **2017**, *38*, 123.

(5) Lehmkühler, F.; Dallari, F.; Jain, A.; Sikorski, M.; Möller, J.; Frenzel, L.; Lokteva, I.; Mills, G.; Walther, M.; Sinn, H., et al. Emergence of anomalous dynamics in soft matter probed at the European XFEL. *Proc. Natl. Acad. Sci. U.S.A* **2020**, *117*, 24110–24116.
